# The Notch Ligand JAG1 Is Required for Sensory Progenitor Development in the Mammalian Inner Ear

**DOI:** 10.1371/journal.pgen.0020004

**Published:** 2006-01-13

**Authors:** Amy E Kiernan, Jingxia Xu, Thomas Gridley

**Affiliations:** The Jackson Laboratory, Bar Harbor, Maine, United States of America; Harvard Medical School, United States of America

## Abstract

In mammals, six separate sensory regions in the inner ear are essential for hearing and balance function. Each sensory region is made up of hair cells, which are the sensory cells, and their associated supporting cells, both arising from a common progenitor. Little is known about the molecular mechanisms that govern the development of these sensory organs. Notch signaling plays a pivotal role in the differentiation of hair cells and supporting cells by mediating lateral inhibition via the ligands Delta-like 1 and Jagged (JAG) 2. However, another Notch ligand, JAG1, is expressed early in the sensory patches prior to cell differentiation, indicating that there may be an earlier role for Notch signaling in sensory development in the ear. Here, using conditional gene targeting, we show that the *Jag1* gene is required for the normal development of all six sensory organs within the inner ear. Cristae are completely lacking in *Jag1-*conditional knockout *(cko)* mutant inner ears, whereas the cochlea and utricle show partial sensory development. The saccular macula is present but malformed. Using SOX2 and p27^kip1^ as molecular markers of the prosensory domain, we show that JAG1 is initially expressed in all the prosensory regions of the ear, but becomes down-regulated in the nascent organ of Corti by embryonic day 14.5, when the cells exit the cell cycle and differentiate. We also show that both SOX2 and p27^kip1^ are down-regulated in *Jag1-cko* inner ears. Taken together, these data demonstrate that JAG1 is expressed early in the prosensory domains of both the cochlear and vestibular regions, and is required to maintain the normal expression levels of both SOX2 and p27^kip1^. These data demonstrate that JAG1-mediated Notch signaling is essential during early development for establishing the prosensory regions of the inner ear.

## Synopsis

Deafness and adult-onset hearing loss are significant health problems. In most cases, deafness or vestibular dysfunction results when the sensory cells in the inner ear, known as hair cells, degenerate due to environmental or genetic causes. In the mammalian inner ear, the hair cells and their associated supporting cells can be found in six different patches that have particular functions related to hearing or balance. Unfortunately, unlike in birds or fish, mammalian hair cells show little ability to regenerate, resulting in a permanent hearing or balance disorder when damaged. Here, the authors show that a protein called JAG1, a ligand in the Notch signaling pathway, is required for the normal development of all six sensory regions in the mammalian inner ear. In ears that lacked JAG1, some of the sensory patches were missing completely, whereas others were small and lacked particular cell types. The authors showed that JAG1 is required by the sensory precursors, progenitor cells that give rise to both the hair cells and the supporting cells. By understanding how the sensory areas develop normally, it is hoped that molecular tools can be developed that will aid sensory regeneration in the mammalian inner ear.

## Introduction

The mammalian inner ear is a complex structure consisting of a coiled cochlea, three orthogonally positioned semicircular canals, a central vestibule, and a dorsally projecting endolymphatic duct and sac. With the exception of the endolymphatic duct and sac, the different parts of the ear all contain sensory organs populated by sensory hair cells and their associated supporting cells. There are three different categories of sensory organs: cristae, located at the base of each semicircular canal; maculae, housed within the central vestibule; and the organ of Corti, which lines the cochlear duct. Only one sensory organ, the organ of Corti, is required for hearing; the other five organs are important for balance. Unfortunately, in mammals, if these regions are damaged due to an environmental or genetic insult, they cannot regenerate, leaving a permanent hearing and/or balance impairment.

Although some progress has been made in understanding how the individual cell types within the sensory areas of the ear are formed [1,2], little is known about the molecular mechanisms that establish the prosensory lineage and how the different sensory organ types are formed. Interestingly, the molecular mechanisms that underlie sensory differentiation in the vertebrate inner ear demonstrate strong parallels with *Drosophila* sense organ development [3–5]. For example, during *Drosophila* external sense organ development, lateral inhibition mediated by Notch signaling is required to restrict the adoption of the sensory organ precursor cell fate, which then gives rise to the entire sensory organ [6–8]. Similarly, in the vertebrate ear, lateral inhibition mediated by Notch signaling appears to be important for restricting the number of cells that can adopt the hair cell fate [9–15]. Lineage analysis has also shown that, at least in the chicken, hair cells and supporting cells arise from a common progenitor [16], consistent with an equipotent epithelium that undergoes lateral signaling to specify cell fates. Unlike in *Drosophila,* which has a single Notch receptor and two ligands (Delta and Serrate/Jagged), in mammals Notch signaling pathway components include four receptors (Notch 1–4) and five ligands (Delta-like [DLL] 1, 3, and 4, and Jagged [JAG] 1 and 2; for reviews, see [8,17–19]). In the mouse, both DLL1 and JAG2 are expressed in nascent hair cells [10,20] and act synergistically during lateral inhibition [15]. Both DLL1 and JAG2 appear to signal through the NOTCH1 receptor [15]. In *Drosophila,* prosensory regions that undergo lateral inhibition are first delineated by expression of members of the *atonal* or *acheate-scute* family of basic helix-loop-helix transcription factors. Further parallels with *Drosophila* have arisen with the finding that an *atonal* homolog, the *Math1* gene, is required for hair cell differentiation [21–23]. However, the situation is not entirely similar to *Drosophila,* as MATH1 does not appear to be required to establish the prosensory regions [22,24]. Instead, it has turned out that another type of transcription factor, the HMG-box factor SOX2, is required for sensory organ formation in the inner ear [25]. This finding does not demonstrate direct parallels with *Drosophila* sense organ formation, since the *Drosophila* SOX2 homologs *SoxNeuro* and *Dicheate* have no known role in peripheral sense organ formation. Instead, both genes play a role in the formation of neural progenitor cells in the central nervous system [26,27]. Consistent with a prosensory role, SOX2 marks sensory progenitors early in development and acts upstream of the *Math1* gene during sensory organ formation in the ear [25]. Interestingly, one of the Notch ligands, JAG1, is expressed early in the prosensory regions of the ear [4,20], indicating that Notch signaling also may play a role in early sensory organ formation. Consistent with this finding, mice heterozygous for *N*-ethyl-*N*-nitrosourea-induced point mutations in the *Jag1* gene show mild sensory organ defects in the ear [28,29]. Unfortunately, embryos homozygous for available mutant alleles of the *Jag1* gene do not survive past embryonic day (E) 11.5 due to vascular defects, precluding an analysis of their inner ears. To circumvent this early lethality, we have created a conditional allele of the *Jag1* gene using Cre/loxP technology. Using the *Foxg1-Cre* mouse line to express Cre recombinase in the early otocyst [30,31], we have disrupted JAG1 function in the ear and show that sensory formation in the inner ear is severely attenuated in these mutants. Analysis of the patterns of hair and supporting cell formation in the *Jag1*-conditional knockout *(cko)* inner ears suggests that fewer progenitors form in *Jag1-cko* inner ears. This result is confirmed by analysis of the prosensory markers SOX2 and p27^kip1^, which are down-regulated as early as E12.5, indicating that the *Jag1* gene acts early during sensory progenitor formation. These data demonstrate an early role for Notch signaling in establishing the sensory progenitors of the inner ear.

## Results

### Creation of a Conditional Allele of the *Jag1* Gene

**Figure 1:**
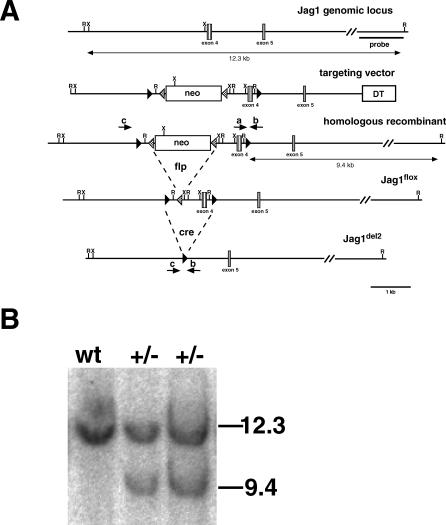
Construction of a Conditional Allele of the *Jag1* Gene (A) Schematic diagram showing the strategy for generating *Jag1^flox^* mice. The targeting vector was designed to insert *loxP* sites (black arrowheads) on either side of exon 4, the DSL domain-encoding exon (white area in exon 4). The neomycin resistance cassette (for positive selection) was flanked by *FRT* sites (gray arrowheads) so that it could later be removed by crossing to *FLPe*-expressing mice. A diphtheria toxin gene was included for negative selection. Dotted lines depict the recombination events that occur when either the FLPe or Cre recombinases are present. Primer positions for genotyping are shown as small black arrows (a, DSLF; b, J1LoxR1; c, J1FlpF1; see Materials and Methods for sequences). DT, diphtheria toxin; R, EcoR1; X, Xba1. (B) Southern blot analysis of EcoRI-digested DNA from ES cells using the external probe shown in (A). Left lane shows the wild-type band (12.3 kb), and the center and right lanes show correctly targeted ES cells that have both a wild-type band and a smaller mutant band (9.4 kb).

**Figure 2:**
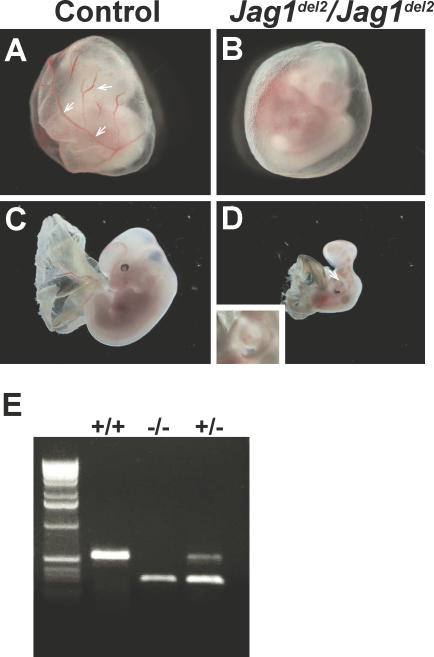
*Jag1^del2^/Jag1^del2^* Embryos Exhibit Vascular Defects and Lethality Consistent with Loss of JAG1 Function (A and B) E10.5 embryos demonstrating the loss of large blood vessels (white arrows in [A]) in the *Jag1^del2^/Jag1^del2^* yolk sacs (B) similar to other *Jag1* loss-of-function mutants. (C and D) E11 embryos demonstrating a small, necrotic *Jag1^del2^/Jag1^del2^* embryo (D). White arrow in (D) indicates an enlarged pericardial sac (enlarged, inset), which is frequently observed in mutants exhibiting cardiovascular defects. RT-PCR results using primers that span exon 4 using RNA extracted from E10.5 control (+) and *ZP3-Cre* deleted embryos (−) (E). The upper band (541 bp) indicates that the wild-type allele is present. The lower bands (286 bp) indicates the *Jag1^del2^* mutant allele that does not contain exon 4.

We created an allele for conditional inactivation of JAG1 function by flanking the Delta-Serrate-Lag2 (DSL) domain-encoding exon (exon 4) of the *Jag1* gene with loxP sites (Figure 1). The DSL domain has been shown to be the region of the DLL and JAG proteins that interacts with Notch family receptors [32,33]; therefore, removing this region of the gene should create a nonfunctional protein. To demonstrate that this allele encodes a nonfunctional protein, we crossed *Jag1^flox^*/+ mice to mice expressing Cre recombinase in the female germline under control of the *Zp3* promoter (*Zp3-Cre* mice); the *Zp3-Cre* mouse strain has been shown to express Cre recombinase in the growing oocyte prior to the completion of the first meiotic division [34]. Female *Zp3-Cre*/+; *Jag1^flox^*/+ offspring were then crossed to male B6 mice to produce offspring that were heterozygous for the deleted region of the floxed allele (designated *Jag1^del2^*/+; see Materials and Methods). Heterozygous *Jag1^del2^*/+ mice were intercrossed, and *Jag1^del2^*/*Jag1^del2^* homozygous offspring were analyzed for defects between E9.5 and E11.5. *Jag1^del2^*/*Jag1^del2^* mutant embryos exhibited the same vascular phenotype we described previously in embryos homozygous for a targeted *Jag1* null allele, *Jag1^del1^* [35]. Specifically, the *Jag1^del2^*/*Jag1^del2^* mutant embryos exhibited yolk sac vascular remodeling defects and cranial hemorrhaging, and often exhibited an enlarged pericardial sac (Figure 2A–2D). All *Jag1^del2^*/*Jag1^del2^* mutant embryos were necrotic by E11.5, and most showed vascular defects by E10.5. RT-PCR of cDNA synthesized from the mutant embryos using primers that span the floxed exon 4 demonstrated that this region was deleted, as expected (Figure 2E). These data demonstrate that deletion of the *Jag1^flox^* allele yields a nonfunctional *Jag1* mutant allele.

### Inactivation of *Jag1* Function within the Ear

**Figure 3:**
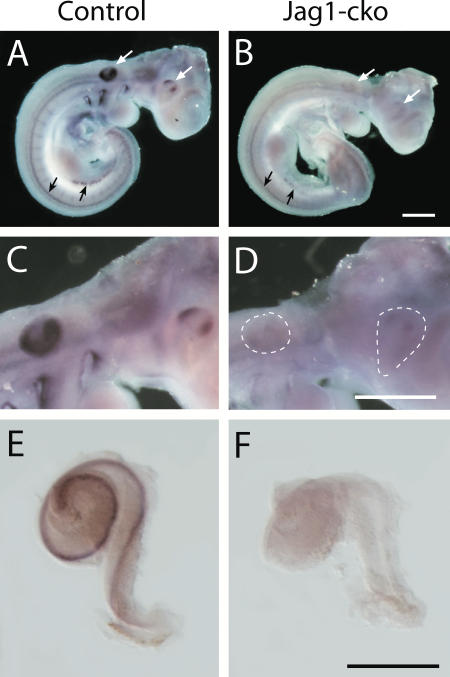
Conditional *Jag1* Inactivation Using the *Foxg1-Cre* Line (A–D) Low- and high-power views of E10 embryos processed for whole-mount in situ hybridization using a *Jag1* exon 4-specific probe. White arrows (A) point to the *Jag1* signal in the otocyst (left arrow) and the eye (right arrow), two structures where Cre recombinase is expressed. In *Jag1-cko* mutants at E10, this signal is either absent or extremely weak. Black arrows (A and B) point to expression in the spinal cord and nephric duct, regions where Cre recombinase expression has not been reported in *Foxg1-Cre* mice. However, expression is consistently weaker in these areas in *Jag1-cko* embryos, indicating that there may be low levels of widespread expression of Cre recombinase in *Jag1-cko* embryos. In (D), the otocyst and the eye are outlined by a dotted line. Very little expression is observed in these regions, consistent with *Foxg1-Cre* expression. (E and F) In situ hybridization of E16.5 cochleae demonstrating *Jag1* expression in wild-type (E) and *Jag1-cko* cochleae (F), where expression is entirely absent. Scale bars = 500 μm.

To disrupt JAG1 function within the inner ear, we crossed *Jag1^flox^*/*Jag1^flox^* mice with mice doubly heterozygous for the *Foxg1-Cre* allele (these mice express Cre recombinase throughout the otocyst, as well as forebrain, eye, and foregut) [30] and the *Jag1^del1^* allele [35]. Offspring with the genotype *Foxg1-Cre/*+; *Jag1^del1^*/*Jag1^flox^* (hereafter designated *Jag1-cko*) survived through E18.5 and were analyzed for inner ear defects. We examined the patterns of Cre-mediated excision in *Jag1-cko* embryos at E10.5 and in cochleae at E16.5–E18.5 by in situ hybridization using a probe that specifically detected the deleted exon 4 (Figure 3). These results showed that expression in the otocyst was weak or absent by E10.5 (Figure 3B). In addition, analysis of cochlear expression at later stages showed no expression at E16.5 (Figure 3F) and E18.5 (unpublished data). These data indicate that, as previously shown for conditional deletion of a *Fibroblast growth factor receptor 1 (Fgfr1)* floxed allele [31], the *Foxg1-Cre* line efficiently deletes the *Jag1^flox^* allele early during inner ear development.

### Malformation of the Inner Ear in *Jag1-cko* Mutants

**Figure 4:**
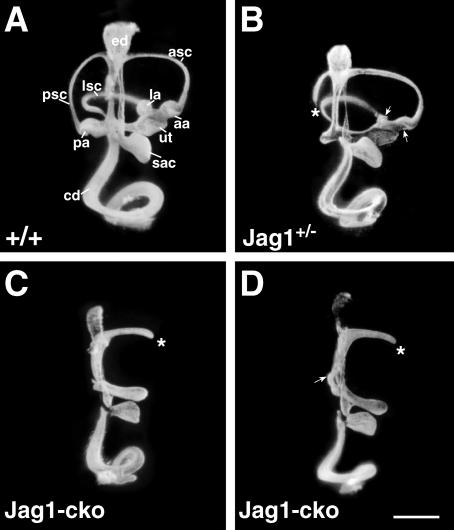
Inner Ear Dysmorphology in *Jag1* ^+*/*−^ and *Jag1-cko* Embryos E15.5 inner ears that have been paintfilled to display their overall morphology. (A) Wild-type inner ear showing normal morphology. Structures are labeled as follows: aa, anterior ampulla; asc, anterior semicircular canal; cd, cochlea duct; ed, endolymphatic duct; la, lateral ampulla; lsc, lateral semicircular canal; pa, posterior ampulla; psc, posterior semicircular canal; sac, saccule; ut, utricle. (B) *Jag1* heterozygote inner ears (either *Jag1^del1^*/+ or *Foxg1-Cre*/+; *Jag1^flox^*/+) display truncated posterior semicircular canals and missing ampullae (asterisk). Arrows point to the anterior and posterior ampullae, which are small compared to the wild-type control (A). (C and D) A much more severe phenotype is observed in *Jag1-cko* animals. There are no ampullae and little semicircular canal development; an asterisk indicates a remnant of the anterior canal. In (D), there is also a remnant of the lateral canal (arrow). The utricle and saccule are smaller and the cochleae are shorter and undercoiled. Scale bar = 500μm.

To examine the morphology of the *Jag1-cko* inner ears, paintfilling of the inner ears of mutants and controls was performed at E15.5 (Figure 4). Results of this analysis showed a severe disruption in the structure of the *Jag1-cko* inner ears compared to their littermate controls (Figure 4C and 4D). Specifically, the semicircular canals were largely absent, with the exception of a portion of the anterior and lateral semicircular canals. In addition, the utricle appeared small, the saccule was misshapen, and the cochlea was undercoiled. In contrast, the parts of the inner ear that are not associated with sensory formation, including the endolymphatic duct and sac and the common crus, appeared relatively unaffected.

### Sensory Defects in *Jag1-cko* Mutant Inner Ears

**Figure 5:**
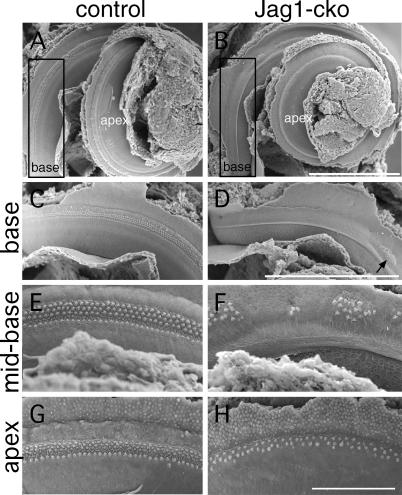
Hair Cell Patterning Defects in the Cochlea Scanning electron micrographs demonstrating the different patterns of hair cell production along the length of the cochlea in *Jag1-cko* embryos. (A–D) Low-power views of the apical and basal cochlear turns. The boxed-in area along the base in (A) and (B) is shown at higher magnification in (C) and (D). Note the absence of hair cells in the base of the *Jag1-cko* cochlea, except for a small patch of cells in the more apical portion (arrow). Scale bars = 500 μm. (E and F) In the midbasal region, more hair cells are observed, but they are arranged in patches, with no clear distinction between inner and outer hair cells. (G and H) In the apical turn, hair cells are continuous but generally arranged in only two rows. Scale bar = 100 μm.

Since the *Jag1* gene is expressed in the sensory areas of the ear, and because the structural malformations observed in *Jag1-cko* mutant inner ears appeared to primarily affect regions of the ear that contained sensory organs, we examined the sensory regions of the ear for defects. We examined the organ of Corti, the sensory organ of the cochlea, at E18.5 by scanning electron microscopy (SEM) (Figure 5). By this stage, all hair cells within the organ of Corti have exited the cell cycle, and most are well-differentiated although not fully mature [36]. Severe hair cell patterning defects were apparent by SEM within the *Jag1-cko* mutant cochleae. This phenotype was most striking in the basal turns of the cochlea, where no hair cell formation was observed (Figure 5D). In the midbasal regions of the organ of Corti, hair cells formed in patches, within which there was no clear formation of rows or distinction between inner and outer hair cells (Figure 5F). More apically, hair cells appeared more continuous along the organ of Corti (Figure 5H). However, although hair cells were present in the apical region, their numbers were clearly reduced; rather than the normal, perfectly ordered four rows of hair cells, there were only two rows of loosely arranged hair cells of indistinct type.

### Abnormal Hair and Supporting Cell Patterns in *Jag1-cko* Inner Ears

**Figure 6:**
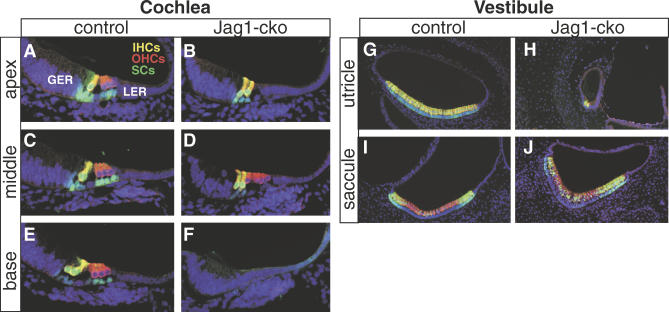
Hair and Supporting Cell Markers Demonstrate Sensory Areas Are Reduced or Absent in the *Jag1-cko* Inner Ear Immunocytochemistry using two markers, myosin VIIA (red; all hair cells) and S100a (green; inner hair cells, Dieter's cells, and inner phalangeal cells) demonstrate patterns of hair and supporting cell production at E18.5 in control and *Jag1-cko* inner ears. (A–F) Sections through the indicated turns of the cochlea. Note the different hair cell patterns in the apical, middle and basal turns of the *Jag1-cko* cochlea. Normal morphology is shown (A) along with labeled structures, as follows: GER, greater epithelial ridge; IHCs, inner hair cells (color-coded yellow); LER, lesser epithelial ridge; OHCs, outer hair cells (color-coded red); SCs, supporting cells (color-coded green). (G–J) Patterns of hair and supporting cell production in the vestibular system. The utricular macula is extremely small with very few hair cells (H) while the saccule (J) shows robust hair and supporting cell production although the shape of the organ is smaller and malformed.

To determine which sensory cell types were differentiating in the *Jag1-cko* mutant cochleae, specific markers were used to identify hair cell and supporting cell subtypes throughout the ear (Figure 6). In the cochlea, we used an antibody against MYO7A to label all hair cells and an antibody against S100A1 to label inner hair cells, Deiter's supporting cells, and inner phalangeal supporting cells [24]. When both markers were used in combination, inner hair cells, outer hair cells, and some supporting cell types could be distinguished (Figure 6A–6F). This analysis showed that in the apex of the cochlea, inner hair cells were present and usually formed as doublets (Figure 6B). Their associated supporting cells, the inner phalangeal cells, were also present. Outer hair cells and their associated supporting cells, the Deiter's cells, were not present in this region. In the middle portions of the cochlea, both inner and occasionally outer hair cells were present, although their patterning was clearly abnormal (Figure 6D). In addition, the tunnel of Corti was not apparent, and there were often doublets of inner hair cells and increased numbers of outer hair cell rows without accompanying Deiter's supporting cells. As shown by SEM, both hair cells and supporting cells were absent in the very basal regions of the cochlea (Figure 6F).

Using the same markers we also examined the vestibular sensory organs in *Jag1-cko* mutant inner ears (Figure 6G–6J). Consistent with the lack of semicircular canal and ampulla formation observed by paintfilling, there was no evidence of crista formation. The *Jag1-cko* utricular macula was extremely small with very few differentiating hair cells (Figure 6H). Surprisingly, the saccule and its macula were only mildly affected in the *Jag1-cko* inner ears (Figure 6J). Hair cell differentiation appeared relatively unaffected, although the entire saccular structure was shaped differently than in the controls, a feature that was also observed in the paintfilled specimens (see Figure 4C and 4D). These data show that all sensory organs within the inner ear are affected to varying degrees in *Jag1-cko* inner ears. However, some sensory organs, such as the cristae, appear to be more sensitive to the loss of JAG1 function.

**Figure 7:**
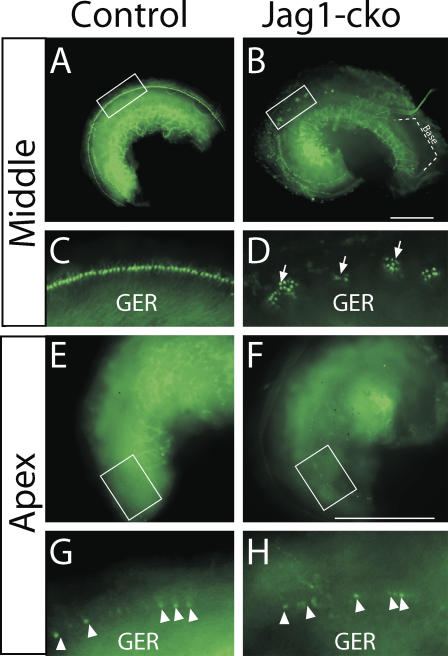
Early Analysis of the Patterns of Differentiation in the *Jag1-cko* Cochlea Indicates the Defects Are Caused by a Failure in the Formation of Sensory Cells and Not Subsequent Degeneration Lectin staining of whole-mount cochlea at E16.5. (A) Normal patterning in wild-type control cochlea. GER, greater epithelial ridge. (B) Both the basal and middle portions of the cochlea are shown, although because it is much longer in the control (A), the very basal portion of the cochlea has been removed. Note the lack of hair cells in the basal portion of the *Jag1-cko* cochlea. (C–H) Boxed-in areas of (A) and (B) are shown at higher magnification in (C) and (D). Arrows in (D) indicate the abnormal patches of hair cells also observed at E18.5. Similarly, the boxed-in regions of (E) and (F) are shown at higher magnification in (G) and (H), demonstrating the few hair cells that are just beginning to differentiate in this region in both the control and the mutant (arrowheads). Scale bars = 500 μm for the corresponding panels.

To examine whether aberrant hair cell patterning in *Jag1-cko* cochleae was due to defects in hair cell formation or in subsequent differentiation, we examined hair cell patterning at an earlier stage (E16.5). Using a lectin that binds to hair cell stereocilia, we examined whether the patterns of hair cell formation at E16.5 looked similar to the patterns at E18.5 (Figure 7). At E16.5 in wild-type cochleae, a gradient of hair cell differentiation was evident (Figure 7A, 7C, 7E, and 7G); in the basal regions both inner and outer hair cells could be recognized (unpublished data), while in the middle regions only inner hair cells were clearly detected by most markers (Figure 7A and 7C). In the more apical regions, little to no hair cell differentiation had taken place by this stage (Figure 7E and 7G). In *Jag1-cko* cochleae, the patterns looked similar to those at E18.5, with patches of hair cells in the midbasal regions (Figure 7B and 7D) and a complete absence of hair cells in the very basal regions (Figure 7B). These data suggest that the *Jag1-cko* mutants have defects in hair cell formation rather than differentiation. In addition, the apical regions in the *Jag1-cko* cochleae did not appear more differentiated than the controls (Figure 7E–7H), arguing against precocious differentiation as an explanation for the reduced numbers of hair cells observed in the mutant cochleae.

### Disrupted Prosensory Development in *Jag1-cko* Inner Ears

**Figure 8:**
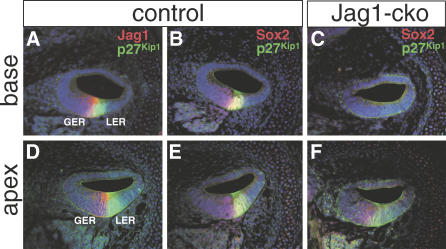
At E14.5 JAG1 Is Expressed Directly adjacent to the Prosensory Domain That Is Disrupted in *Jag1-cko* Inner Ears Immunocytochemistry at E14.5 using two markers of the prosensory domain, Sox2 and p27^Kip1^, in combination with JAG1 in both the basal and apical turns of the cochlea. Note that the JAG1 domain (red) does not overlap the p27^Kip1^ domain (green) (A and D), whereas the SOX2 domain does largely overlap with p27^Kip1^ (yellow) (B and E). Both SOX2 and p27^Kip1^ are down-regulated in the *Jag1-cko* cochlea (C and F), although there is weak expression of both markers in the apex (F). GER, greater epithelial ridge; LER, lesser epithelial ridge.

To determine how the JAG1 ligand functions during sensory development, we used several markers of the prosensory domain, including p27^kip1^ and SOX2, and examined their expression patterns in both wild-type and *Jag1-cko* mutant cochleae (Figure 8). At E14.5, the majority of hair cells and supporting cells in the organ of Corti have completed their final division, and hair cells are beginning to differentiate in the basal portions of the cochlea [36]. p27^kip1^, a cell-cycle inhibitor, is required for the cochlear sensory progenitors to exit the cell cycle on time, and is an established marker of the prosensory domain in the cochlea [22,37]. p27^kip1^ begins to be expressed in a discrete domain within the cochlea as the hair cells and supporting cells exit the cell cycle around E13.5 to E14.5 (Figure 8A, 8B, 8D, and 8E). Recently it has been shown that the SRY-related transcription factor SOX2 is required for establishment of the prosensory regions in the inner ear [25]. Using fluorescence immunocytochemistry double labeling, we examined the relationship between these markers and JAG1 protein expression in both wild-type and *Jag1-cko* cochleae. As previously reported [22], JAG1 was not expressed within the prosensory domain as assessed by p27^kip1^ expression at E14.5, but instead was expressed immediately adjacent (possibly with some slight overlap) in the inner (neural) portion of cochlea (Kölliker's organ; Figure 8A and 8D). In contrast, SOX2 did show a largely overlapping domain with p27^kip1^ (Figure 8B and 8E), as originally described [25]. However, the SOX2 expression domain was slightly larger than the p27^kip1^ domain, extending into Kölliker's organ and overlapping with the JAG1 domain. Despite the fact that JAG1 was not expressed within the prosensory domain at E14.5, both p27^kip1^ and SOX2 expression was absent in the basal regions of the cochlea (Figure 8C), indicating that prosensory formation is already disrupted in these ears. In the apex, weak expression of both markers was observed (Figure 8F), consistent with the fact that some sensory differentiation occurs in this region of the *Jag1-cko* cochlea.

**Figure 9:**
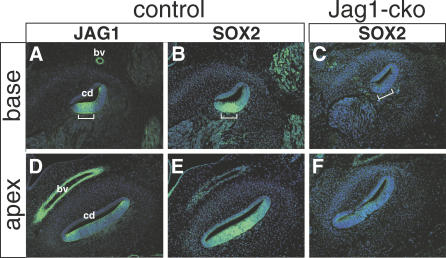
At E12.5 JAG1 Is Expressed within the Prosensory Domain and SOX2 Expression Is Down-Regulated within This Domain in *Jag1-cko* Cochleae (A, B, D, and E) Alternate sections from a control embryo processed for immunocytochemistry using antibodies against either JAG1 or SOX2. Note the similar domain occupied by both JAG1 and SOX2 in the base of the cochlea (A and B; brackets). In the apical region, the two proteins are not colocalized (D and E). SOX2 is not expressed in the basal portions of the *Jag1-cko* cochlea (C) and shows only weak expression in the apex (F). bv, blood vessel; cd, cochlear duct.

In order to determine if JAG1 is ever expressed in the prosensory region of the cochlea, we examined an earlier age (E12.5) and compared the JAG1 domain to the SOX2 domain (since p27^Kip1^ is not expressed in the inner ear prior to E13.5 to E14.5). Adjacent sections from both wild-type and *Jag1-cko* cochleae were immunostained to detect either JAG1 or SOX2 protein (Figure 9). This analysis showed that in the basal regions of the wild-type cochlea, where sensory progenitors were still dividing, JAG1 expression did overlap with the SOX2 domain (Figure 9A and 9B), indicating that JAG1 is initially expressed within the prosensory domain. However, in the apical regions, where the sensory precursors have ceased dividing, expression of JAG1 and SOX2 did not overlap (Figure 9D and 9E). In the *Jag1-cko* cochlea, SOX2 was absent from the basal regions and significantly down-regulated in the apical regions (Figure 9C and 9F). These data demonstrate that JAG1 is expressed within the prosensory domain of the cochlea at early stages, and that, in the absence of JAG1 function, sensory formation is disrupted prior to cell cycle exit and differentiation of sensory hair cells and nonsensory supporting cells.

**Figure 10:**
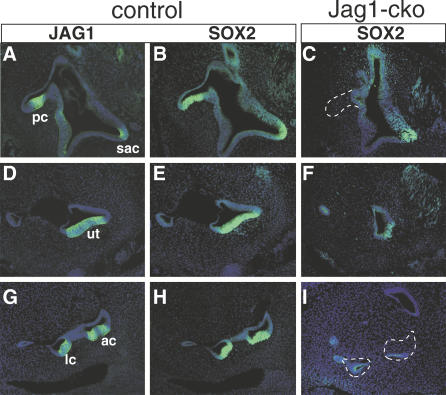
JAG1 and SOX2 Mark the Prosensory Regions of the Vestibule, and SOX2 Expression Correlates with Impaired Sensory Formation in the *Jag1-cko* Vestibule (A and B, D and E, G and H) Alternate sections demonstrating either JAG1 or SOX2 expression in the vestibular regions of control inner ears. (C, F, I) Similar sections through the *Jag1-cko* inner ear demonstrating SOX2 expression. Dotted lines indicate regions where the cristae and ampullae are missing in the *Jag1-cko* inner ear. ac, anterior cristae; lc, lateral cristae; pc, posterior cristae; sac, saccular macula; ut, utricular macula.

We also compared JAG1 and SOX2 expression in the vestibular regions of the inner ear in both wild-type and *Jag1-cko* mutant embryos. JAG1 and SOX2 exhibited largely overlapping expression domains that corresponded to the locations of the five sensory organs in the vestibular portion of the ear (Figure 10). The two expression domains only differed significantly in the anterior and posterior cristae, where JAG1 expression had a negative patch in the middle of its expression domain, whereas SOX2 expression did not show this same patch (Figure 10A, 10B, 10G, and 10H). The JAG1-negative region may correspond to the eminentia cruciatum, a nonsensory region present in the middle of both the anterior and posterior cristae, although it is not clear why SOX2 would be expressed there. In the *Jag1-cko* vestibular sensory patches, SOX2 expression was consistent with the patterns of sensory differentiation observed at E18.5. For example, the *Jag1-cko* saccule displayed fairly normal SOX2 expression (Figure 10C), consistent with the almost normal development of the saccular macula. In contrast, SOX2 expression in the utricle was very weak and the expression domain was much smaller than in controls (Figure 10F), consistent with the severe disruption of differentiation of the utricular macula in *Jag1-cko* inner ears. There was no SOX2 expression in the *Jag1-cko* cristae, and in fact the entire ampullae appeared to be missing or severely disrupted even at this early stage (Figure 10C and 10I; dotted line regions), consistent with the lack of cristae and ampullae observed at later stages.

## Discussion

We have demonstrated that Notch signaling, mediated by the JAG1 ligand, is required early in development for the formation of the sensory regions of the ear. By comparing expression of JAG1 to two markers of the prosensory domain, SOX2 and p27^kip1^, we have shown that JAG1 marks all prosensory regions of the ear from early time points (E12.5), but becomes down-regulated in the organ of Corti by E14.5, when the sensory progenitors exit the cell cycle and begin differentiating into hair cells and supporting cells. Both SOX2 and p27^kip1^ are down-regulated in the affected prosensory regions of the *Jag1-cko* inner ear, demonstrating that JAG1 is necessary for the development of early sensory progenitors in the inner ear.

### Distinctive Patterns of Hair Cell Formation in *Jag1-cko* Inner Ears Suggest Progenitor Cell Numbers Are Reduced

One intriguing result from our studies was that the six sensory regions were not equally affected by the loss of *Jag1* function. For example, in the *Jag1-cko* vestibular system, the cristae were lacking altogether, and only a small number of hair cells differentiated in the utricular maculae. In contrast, the saccular maculae exhibited little disturbance in hair cell formation, although the overall shape of the organ was abnormal. In the *Jag1-cko* cochlea, hair cell differentiation patterns varied based on their apical or basal location. For example, in the apical regions of the cochlea only inner hair cells formed, and these were often arranged in multiple rows rather than the normal single row. In the middle and midbasal turns of the cochlea, patches of hair cells with nonsensory intervening regions were frequently observed. Within these patches, outer hair cells were sometimes present, although the patterning was abnormal and S100A1-labeled Dieter's cells were not present. In the very basal regions of the cochlea, neither hair cells nor supporting cells were present.

The patches of hair cells found in the basal regions of the cochlea and the differential effect of the mutation on the basal and apical portions of the cochlea were particularly interesting, as similar defects have been found in at least two other mouse mutants of genes known to play a role in the generation of the sensory precursors of the ear. For example, both a hypomorphic allele and a conditionally deleted allele of the *Fgfr1* gene exhibited patches of hair cells in portions of the cochlea [31]. Similar to the *Jag1-cko* phenotype, these patches in the *Fgfr1* conditional mutants contained mostly inner hair cells that were often arranged in multiple rows, with very few outer hair cells. Unlike the *Jag1-cko* phenotype, *Fgfr1* function was required only in the cochlea. Another mouse mutant, a hypomorphic allele of the *Sox2* gene (yellow submarine; *Sox2^ysb^*), also displayed patches of hair cells in the basal portions of the cochlea and a milder phenotype in the apical regions of the cochlea [25]. More similar to the *Jag1-cko* phenotype, SOX2 was required for both the cochlear and vestibular sensory regions [25]. The finding that primarily inner hair cells differentiate in these mutant cochleae may be due to the fact that inner hair cells are the first to differentiate [10,37], suggesting that if there are reduced numbers of progenitor cells, they would likely differentiate as inner rather than outer hair cells. Similarly, the milder phenotype in the apical regions of these mutants may be due to the fact that cells exit the cell cycle earliest in the apex [36]. This may mean that, if there are reduced numbers of progenitor cells, they would reside in the apical rather than the basal portions of the cochlea.

The multiple rows of inner hair cells observed in *Jag1-cko* and other mutants could be explained by a number of different scenarios. One possibility is that the multiple rows are not a result of actual increases in inner hair cell numbers, but rather are caused by defects in their eventual arrangement due to the shorter *Jag1-cko* cochlea. Recent studies of mouse mutants with defects in planar cell polarity and convergent extension (a term referring to the intercalation of cells, leading to growth of tissue in one dimension in the absence of proliferation) indicate that multiple rows of hair cells can be obtained in this way and are frequently observed in the apical regions of the cochlea [38–41]. An alternative possibility is that the multiple rows are a result of a second, later function of the JAG1 ligand, distinct from its prosensory role described here. A third possibility is that the down-regulation of p27^kip1^, a protein that inhibits continued proliferation of the precursor cells in the cochlea, leads to continued cell division of the remaining sensory progenitors, ultimately resulting in excess numbers of inner hair cells in the regions where they form. Taken together, these data suggest that sensory progenitors are reduced in the *Jag1-cko* inner ears and that Notch signaling, fibroblast growth factor signaling, and the transcription factor SOX2 all act in either common or parallel pathways involved in the production of sensory progenitors in the inner ear.

### A Prosensory Role for Notch in the Ear

An examination of early prosensory markers, including p27^Kip1^ and SOX2, demonstrated that prosensory establishment is disrupted in *Jag1-cko* inner ears, consistent with the suggestion that progenitors are reduced in these mutants. Our data show that JAG1 plays an early prosensory role in ear development, quite unlike the role played later during development by the other Notch ligands, DLL1 and JAG2, which are involved in lateral signaling and differentiation [10,15]. These data are consistent with an early role for Notch signaling in progenitor cell maintenance in the inner ear. In a number of other systems, including the nervous system and more recently in the intestinal epithelium, it has been demonstrated that Notch signaling is involved in maintaining cells in an undifferentiated state [42–46]. In the mammalian nervous system it has been shown that loss of Notch signaling leads to premature differentiation and a reduction in the progenitor pool [42]. Consistent with these findings, in vitro studies have demonstrated that the frequency of neurosphere production was reduced in Notch signaling mutants [47,48], indicating a loss of stem cell potential. Moreover, studies have also shown that Notch signaling promotes radial glial identity, a cell type that has been shown to act as a progenitor cell in the central nervous system [49–52]. Our results suggest that, similar to the nervous system, Notch signaling via JAG1 is important for sensory precursor formation or maintenance in the inner ear. However, unlike the nervous system, we see no evidence for precocious differentiation, suggesting instead that JAG1 may affect the specification, survival, or proliferative capacity of the sensory precursors.

Recent evidence from the chick indicates that JAG1 may be important for the initial sensory specification events. By expressing a constitutively active form of Notch (Notch1-ICD), Daudet et al. [14] demonstrated that ectopic sensory patches could be induced, indicating that early Notch signaling may be important for the induction of sensory areas, and not just for their maintenance. However, it should be noted that ectopic sensory areas formed only in certain areas of the ear, indicating that some sensory competence is required for this effect. A similar result was obtained by overexpressing an activated form of β-catenin, an essential component of the canonical Wnt signaling pathway, in the chicken inner ear [53]. As in the Notch1-ICD studies, ectopic sensory regions were obtained, but again, only in certain regions of the ear. However, unlike the Notch gain-of-function studies, overexpression of β-catenin also led to a change in sensory region character (i.e., cochlear to vestibular), indicating that Wnt signaling governs not only whether a sensory region will form but also the type of sensory region that will form. In *Drosophila,* interactions between Notch and Wingless, a member of the Wnt family of signaling molecules, are well established [54,55], and evidence of an interaction has begun accumulating in vertebrates as well [45,56,57]. Bone morphogenetic protein (BMP) signaling may also be important for sensory formation, particularly for the sensory cristae, as BMP4 has been shown to mark the mouse cristae from very early in development [58]. Experiments in the chicken have shown that blocking BMP signaling sometimes leads to disturbances in sensory development [59]. Taken together, these data indicate that, based on expression patterns, previous studies, and the evidence presented here, JAG1 is the ligand responsible for the prosensory function of the Notch pathway in the ear. Furthermore, the Notch pathway likely interacts with other signaling pathways such as the Wnt, FGF, and BMP pathways to create sensory organs of the proper size, organization and character.

### Sensory Formation Still Occurs in *Jag1-cko* Inner Ears

One somewhat puzzling question is that, if JAG1 is important for sensory progenitor development, why does any sensory formation occur in *Jag1-cko* inner ears? One possibility is that another Notch ligand is compensating for the loss of JAG1 function. This explanation seems unlikely since none of the other Notch ligands shows a similar expression pattern to JAG1 in the ear. For example, both the *Dll1* and *Jag2* genes are expressed in nascent hair cells after they exit the cell cycle and begin differentiating. However, in addition to hair cell expression, there is also early expression of the *Dll1* gene in the anteroventral portion of the otocyst at about E10.5 [4,20], that likely overlaps with at least part of the JAG1 domain (see Figure 2) [60]. This expression domain has previously been thought to be related to the formation of the neuroblasts that delaminate from the otic epithelium and later differentiate into the neurons that will innervate the hair cells [4]. It has been shown in zebrafish that correct neuroblast formation requires Notch-mediated lateral signaling [9]; however, in mammals it has not been shown definitively that this is the role that the *Dll1* gene plays at early stages. This leaves open the possibility that this early domain of DLL1 expression may be at least partially involved in prosensory specification, similar to the JAG1 expression domain.

### Nonsensory Defects in *Jag1-cko* Inner Ears

In addition to the defects in sensory formation in the *Jag1-cko* inner ears, the mutant inner ears also exhibited nonsensory defects. Specifically, the semicircular canals were largely absent, with the exception of portions of the anterior and lateral canals. In addition, all three ampullae were absent, the utricle was small, and the cochlea was undercoiled. Based on recent studies, it is likely that these defects are secondary to the sensory defects. For example, it has been shown that FGFs expressed in the sensory cristae promote semicircular canal formation through up-regulation of BMP2 [61]. Thus, loss of the cristae would be expected to have a severe affect on canal formation. Emerging evidence from mouse mutants has demonstrated that genes involved in sensory formation result in severely malformed inner ears. For example, mutations in the *Sox2* gene lead to malformations very similar to those described here in *Jag1-cko* mutants. The inner ears of embryos homozygous for two different mutant alleles of *Sox2, Sox2^lcc/lcc^* and *Sox2^ysb/ysb^,* showed disrupted canal formation; smaller utricular and saccular compartments; and thinner, undercoiled cochleae [25]. In addition, FGF10 mouse knockouts also showed disrupted cristae development associated with loss of canal structures [62]. However, unlike the canal structures, cochlear formation does not appear to be strictly dependent on development of the organ of Corti, as a cochlea, albeit short and thin, will form in the absence of any sensory formation [25]. However, normal cochlear length appears to be dependent on sensory formation, at least partially through convergent extension. Recently, a number of genes have been found in the cochlea that lead to defects in planar cell polarity as well as a shortened cochlea, presumably because of defects in convergent extension [38,41]. Therefore it is likely that the shortened cochlea observed in *Jag1-cko* mutants is at least partially a result of failure of convergent extension caused by a reduction in the number of sensory precursors.

The data presented here demonstrate that the *Jag1* gene is required for sensory precursor development in the inner ear. Further studies are required to establish the exact role that JAG1-mediated Notch signaling plays in early sensory progenitors, and also its relationship to the roles played by FGF signaling and SOX2 expression. Understanding how the sensory precursors form is an important prerequisite for regeneration studies that may provide molecular tools to treat hearing loss and vestibular disorders [63].

## Materials and Methods

### Construction of the *Jag1^floxneo^* allele.

To construct the *Jag1^floxneo^* allele, bacterial artificial chromosome clones containing the *Jag1* genomic locus were isolated from a RPCI-22 (129S6/SvEvTac) mouse bacterial artificial chromosome library (filters obtained from Research Genetics) by hybridization to a 1.8-kb mouse *Jag1* cDNA probe. To make the shorter 5′ homology region of the targeting vector, a 2.2-kb KpnI fragment upstream of exon 4 was isolated, blunt-ended, and subcloned into the SmaI site of a modified pBS vector that contained a loxP-FRT-PGK*neo*-FRT cassette. A 1.5-kb KpnI fragment that contained exon 4 was also subcloned into the loxP-FRT-PGK*neo*-FRT cassette. To construct the longer 3′ homology region, a 3.5-kb KpnI-SmaI fragment containing exon 5 was blunt-ended and subcloned into the EcoRV site of another modified pBS vector that contained a single loxP site. A 3.5-kb SmaI-SalI fragment from this construct was then cloned into the SmaI-XhoI site of a pKO 905 vector containing a diphtheria toxin gene for negative selection. A 5.7-kb SalI-NotI fragment from the loxP-FRT-PGK*neo*-FRT construct was then cloned into the SalI-NotI sites of the pKO 905 vector containing the 3′ homology region to generate the final *Jag1^floxneo^* targeting vector (see Figure 1).

### Generation of *Jag1^flox^* mice.

The *Jag1^floxneo^* targeting construct was linearized with Not1 and electroporated into CJ7 embryonic stem (ES) cells, as described previously [64]. DNA from 288 ES cell clones was screened by PCR using an internal/external primer set, and positive clones were then confirmed by Southern blot by probing EcoRI-digested DNA with an external 1.7-kb Stu1-EcoRI fragment located 3′ to the targeting construct (see Figure 1). This probe also detected partial recombination events in which the distal loxP site was lost; in these cases a slightly larger fragment (11 kb rather than 9.3 kb) was obtained (see Figure 1A). The presence of the distal loxP site was further confirmed by PCR using primers that flanked the loxP site (DSLF and J1LoxR1; see below for sequences). Correctly targeted clones were injected into C57BL/6J (B6) blastocysts, and chimeric mice were obtained. Chimeric male mice were mated to B6 females and the agouti progeny were assayed for the presence of the *Jag1^floxneo^* allele by PCR using *Jag1^floxneo^* specific primers. *Jag1^floxneo^*/+ mice were intercrossed to create homozygous *Jag1^floxneo^*/*Jag1^floxneo^* offspring. Homozygous *Jag1^floxneo^*/*Jag1^floxneo^* mice appeared normal and healthy, suggesting that the neomycin resistance cassette (PGK*neo*) did not adversely affect *Jag1* expression. To control for any possible effects from the presence of the PGK*neo* cassette, the FRT-flanked PGK*neo* cassette was deleted by mating *Jag1^floxneo^* mice to *FLPe*-recombinase expressing mice (*Gt[ROSA]26Sor ^tm1(FLP1)Dym^;* Jackson Laboratory, Bar Harbor, Maine, United States) to produce *Jag1^flox^* mice. Both *Jag1^floxneo^* and *Jag1^flox^* mice were used in these experiments, and no differences in the resulting phenotype were observed. To differentiate the deleted form of this allele from our previously reported *Jag1* null mutant allele *(Jag1^del1^)* [35], we designate the *Jag1* allele generated by Cre recombinase-mediated deletion of the *Jag1^flox^* or *Jag1^floxneo^* alleles the *Jag1^del2^* allele.

### Mouse husbandry and genotyping.

*Foxg1-Cre* mice ([30]; gift of Rob Burgess) were maintained on an outbred Swiss Webster background. *ZP3-Cre* mice ([34]; gift of Mimi de Vries and Barbara Knowles) were maintained on a B6 background. Typically, males that were heterozygous for both a *Foxg1-Cre* allele and our previously constructed *Jag1* null allele *(Jag1^del1^)* [35] (maintained on a B6 background), were crossed to *Jag1^flox^*/*Jag1^flox^* females that were maintained on a B6/129 background. Mice of the genotypes *Foxg1-Cre/*+; *Jag1^del1^*/*Jag1^flox^* and *Foxg1-Cre/*+; *Jag1^del1^*/*Jag1^floxneo^* were used interchangeably, and are designated as *Jag1-cko* mice in this report.

To genotype the *Jag1^flox^* mice, the primers used were: DSLF, 5′-TCAGGCATGATAAACCCTAGC-3′ (forward) and J1LoxR1, 5′-CTACATACAGCATCTACATGC-3′ (reverse); these primers flank the 5′ LoxP site. To genotype for CRE-mediated recombination, a primer upstream of the 3′ LoxP site was used: J1FlpF1, 5′-CAGGTTGAGTGCTGACTTAG-3′, along with the J1LoxR1 reverse primer. To genotype for the *Foxg1-Cre* and *ZP3-Cre* alleles, *Cre*-specific primers were used: Cre1, 5′-TGATGAGGTTCGCAAGAACC-3′ (forward) and Cre2, 5′-CCATGAGTGAACGAACCTGG-3′ (reverse). *Jag1^del1^* primers were as follows for the mutant: JGKO1, 5′-TCTCACTCAGGCATGATAAACC-3′ (forward) and SOL1, 5′-TGGATGTGGAATGTGTGCGAG-3′ (reverse). A different reverse primer, JGKO2, 5′-TAACGGGGACTCCGG ACAGGG-3′ was used to detect the wild-type allele. Littermates (wild type, *Jag1^del1^/*+ or *Jag1^flox^/*+) were used as controls for all experiments.

### Paintfilling and scanning electron microscopy.

The paintfilling of the *Jag1-cko* inner ears was performed at E15.5. The technique was performed as previously described [28]. Inner ears were prepared for SEM as described previously using a version of the osmium tetroxide-thiocarbohydrazide method [65]. Specimens were examined with a Hitachi 3000N scanning electron microscope (Hitachi, Tokyo, Japan).

### Immunohistochemistry and lectin staining.

For immunohistochemistry, embryonic heads were bisected and fixed for 1–2 h in 4% paraformaldehyde in PBS. Half heads were embedded in paraffin wax, and immunocytochemistry was performed on standard 7-micron sections. Antibodies used included anti-Myo7a (1:1,000; gift of A. EL-Amraoui and C. Petit), anti-p27^kip1^ (1:100; Neomarkers, Stratech, Soham, Cambridgeshire, United Kingdom), anti-SOX2 (1:2,000; Chemicon, Temecula, California, United States; AB5603), anti-JAG1 (1:100; Santa Cruz Biotechnology, Santa Cruz, California, United States; H-114) and anti-S100A1 (1:50; Dako, Glostrup, Denmark). For all antibodies used, an antigen retrieval step was performed by boiling the sections for 10 min in 10 mM citric acid. Secondary antibodies used were either Alexa-Fluor 488 or 546 goat anti-mouse or rabbit (1:400; Invitrogen, Carlsbad, California, United States). Slides were coverslipped in Vectashield HardSet Mounting Medium with DAPI (Vector Laboratories, Burlingame, California, United States). Lectin staining was performed using the *Griffonia simplifonia* I lectin (Vector Laboratories) essentially as described [15].

### In situ hybridization and RT-PCR.

Since the *Jag1^del2^* mutant allele creates an in-frame deletion of the DSL domain, an in situ probe was designed for detection of the floxed region of the *Jag1^flox^* allele by in situ hybridization. The probe was created by amplifying a 433-bp product encompassing exon 4 (primers: J1-420F, 5′-CGACCGTAATCGCATCGTAC-3′ and J1-853R, 5′-ATGCACTTGTCGCAGTACAG-3′) and subcloning the product into the PCR II vector (Invitrogen). For whole-mount embryos in situ, embryos were fixed overnight in 4% paraformaldehyde. In situ hybridization was performed essentially as described [66]. For cochlear in situ, inner ears were dissected from the head and fixed overnight in 4% paraformaldehyde. After washing in PBS, the bony shell and stria were removed from the cochleae and the samples were dehydrated in methanol. In situ hybridization was performed as described [67], with the exception of the posthybridization washes, which were done according to [68]. For RT-PCR, total RNA was extracted from the E10.5 control and *Jag1^del2^/Jag1^del2^* embryos, using an RNAeasy kit (Qiagen, Valencia, California, United States) and following the manufacturer's instructions. First-strand cDNA synthesis was performed using the AMV reverse transcriptase (Promega, Madison, Wisconsin, United States) with specific primer J1-961R (5′-AGTCCCACAGTAATTCAGATC-3′). Products were amplified from cDNA using primers that flanked exon 4 (J1-420F, 5′-CGACCGTAATCGCATCGTAC-3′ and J1-961R).

## Abbreviations

BMP: bone morphogenetic protein
cko: conditional knockout
DLL: Delta-like
DSL: Delta-Serrate-Lag2
E[number]: embryonic day [number]
ES: embryonic stem
FGF: fibroblast growth factor
JAG: Jagged
SEM: scanning electron microscopy
